# Childhood Cognitive Ability and Age-Related Changes in Physical Capability From Midlife: Findings From a British Birth Cohort Study

**DOI:** 10.1097/PSY.0000000000000482

**Published:** 2017-06-09

**Authors:** Rachel Cooper, Marcus Richards, Diana Kuh

**Affiliations:** From the Medical Research Council Unit for Lifelong Health and Ageing at UCL, London, United Kingdom.

**Keywords:** age-related decline, cognition, life course, neurodevelopment, physical capability, **CI** = confidence interval, **NSHD** = National Survey of Health and Development, **RRR** = relative-risk ratio, **SD** = standard deviation

## Abstract

**Objective:**

The aim of the study was to test the hypothesis that higher childhood cognitive ability is associated with reduced risk of decline in physical capability in late midlife.

**Methods:**

Participants were 1954 men and women from the Medical Research Council National Survey of Health and Development with complete data on cognitive ability at age of 15 years and measures of grip strength and chair rise speed at ages of 53 and 60 to 64 years. Using multinomial logistic regression, associations of childhood cognitive ability with categories of change in grip strength and chair rise speed (i.e., decline, stable high, stable low, reference) were investigated. Adjustments were made for potential confounders from early life and adult mediators including health behaviors, educational level, and cognitive ability at age of 53 years.

**Results:**

Higher childhood cognitive scores were associated with reduced risks of decline in grip strength and chair rise speed, for example, the sex-adjusted relative-risk ratio of decline (versus reference) in grip strength per 1SD increase in childhood cognitive score was 0.82 (95% confidence interval = 0.73–0.92). Higher childhood cognitive scores were also associated with reduced risk of stable low and increased likelihood of stable high chair rise speed.

**Conclusions:**

These findings suggest that childhood cognitive ability may be related to decline in physical capability in late midlife. A number of life course pathways are implicated, including those linking childhood and adult cognitive ability. Future research aiming to identify new opportunities to prevent or minimize age-related declines in physical capability may benefit from considering the potential role of neurodevelopmental as well as neurodegenerative pathways.

## Abstract 2

Supplemental digital content is available in the text.

## INTRODUCTION

There is increasing research interest in the influence of neurodegeneration on age-related declines in mobility and other aspects of physical capability (1,2) and a growing body of empirical evidence highlighting its importance (3). From a life course perspective, neurodevelopment as well as neurodegeneration may underlie this physical decline (4,5). If so, interventions that positively affect neurodevelopmental pathways may, in addition to their benefits in earlier life, prevent, delay, or minimize declines in physical capability later in life.

A few existing studies have examined associations between cognitive ability in earlier life, a commonly used marker of neurodevelopment, and objective measures of physical capability including walking speed, grip strength, and chair rise time at mean ages of assessment from 50 to 79 (4,6–10). Most of these studies present evidence of association between higher cognitive scores in earlier life and higher levels of physical capability in mid to late adulthood. However, associations have not always been consistently found across all measures of physical capability investigated (4,8). In addition, all previous analyses that have examined objective measures of physical capability have done so at only one time point and so cannot distinguish between the potential influences of neurodevelopment on the levels of physical capability achieved by midlife and longer-term influences on subsequent age-related declines.

Although it may generally be expected that early life factors will be more strongly associated with peak levels of physical capability achieved by midlife than with subsequent age-related declines, there is currently insufficient empirical evidence to formally assess this. In addition, there are reasons to expect that childhood cognitive ability may have longer-term associations with age-related changes in physical capability. Firstly, cognitive ability in childhood represents the starting point of a lifelong cognitive pathway, and as higher childhood cognitive ability has been associated with reduced risk of decline in cognitive ability in midlife (11), this may have positive implications for physical capability in later life. Related to this, higher childhood cognitive ability may reflect greater levels of neurophysiological reserve (12,13). If so, even when challenges to physical capability have accumulated across the central and peripheral nervous systems, these may be less likely to reach sufficient levels to precipitate decline in those with higher childhood cognitive ability than in those with lower ability. Finally, childhood cognitive ability is associated with cumulative exposure to a number of extrinsic factors across life (13) shown to be associated with age-related declines in physical capability (14–16).

We thus aimed to extend the existing evidence base by using data from a large nationally representative British birth cohort to test the hypothesis that higher childhood cognitive ability is associated with reduced risk of decline in objective measures of physical capability during late midlife.

## MATERIALS AND METHODS

The Medical Research Council National Survey of Health and Development (NSHD) is a socially stratified sample of 5362 singleton births (2547 males and 2815 females) that took place in 1 week of March 1946 in mainland Britain, with regular follow-up and high participation rates across life (17,18). During two waves of data collection, in 1999 (at 53 years) and 2006–2010 (at 60–64 years), physical capability was assessed using performance-based measures.

Of the original 5362 participants, 3673 were eligible for inclusion in the data collection at age of 53 years. Of these participants, 3035 were successfully contacted and 2984 (81%) received a home visit from a trained nurse. Of the 2327 participants not eligible for inclusion at age of 53 years, 469 had previously died, 948 had refused to participate, 580 were living abroad, and 330 could not be traced. At ages of 60 to 64 years, 2856 eligible participants (those known to be alive, living in England, Scotland, or Wales, and who had not permanently refused to participate) were invited for assessment at one of six clinical research facilities or to be visited by a research nurse at home of whom 2229 (78%) were assessed (1690 at a clinical research facility and 539 at home). Of the 2506 participants not contacted at ages of 60–64 years, 778 had died (309 since age of 53 years), 594 had refused to participate, 578 were living abroad, and 556 could not be traced.

Relevant ethical approval was provided by the North Thames Multi-Centre Research Ethics Committee in 1999 and by the Central Manchester Local Research Ethics Committee and the Scotland A Research Ethics Committee in 2006–2010. All participants provided informed consent.

### Physical Capability at Ages of 53 and 60–64 Years

Grip strength and chair rise time were assessed at ages of 53 and 60–64 years by trained nurses using standardized protocols. At both ages, grip strength was measured isometrically using a Nottingham electronic handgrip dynamometer. To ensure comparability between ages, the highest value achieved of four measures recorded at age of 53 years (two in each hand) and the highest of the first four values (from a total of three in each hand) recorded at ages of 60–64 years were used in analyses. At both ages, chair rise time was measured, using a stopwatch, as the time taken to rise from a sitting to a standing position with straight back and legs and then sit down again ten times as fast as possible. For high scores to indicate good performance, chair rise speed was calculated by dividing the number of rises (i.e., 10) by the time taken (in minutes). Nurses recorded if a study participant was unable or unwilling to perform either test and the reason for this.

To avoid some of the important limitations inherent in modeling change continuously when data are only available at two time points (19,20), we decided a priori to identify categories of change and to model these as our main outcomes. After careful consideration of a number of different approaches, we chose to distinguish between the following four groups: a group experiencing decline; a group with relatively high levels at baseline, which are maintained; a group with relatively low levels at baseline who remain low; and a reference group who maintain physical capability levels within a “normal” range (14). Participants were assigned to one of these four outcome groups for each measure based on the changes in their sex-specific standard deviation scores of grip strength and chair rise speed (categorized as: < −1SD; −1 to 1 SD; >1SD) between ages of 53 and 60–64 years. The groups were defined as the following: (1) decline (change in SD score between ages of 53 and 60–64 years from >1SD to ≤1SD or from −1 to 1SD to < −1SD); (2) stable high (SD score >1 at both ages); (3) stable low (SD score < −1 at both ages); and (4) reference (all other combinations of cross-tabulated SD scores) (see supplemental digital content Table S1, Supplemental Digital Content 1, http://links.lww.com/PSYMED/A396). These categorizations were derived in the samples with valid measures at both ages. Participants with valid values at age of 53 years who were unable to complete the test for health reasons at ages of 60–64 years were added to the “decline” category and participants who were unable to complete the test for health reasons at both ages were added to the “stable low” category.

### Childhood Cognitive Ability

At age of 15 years, study participants were asked to complete the following three cognitive tests: the Heim AH4 test, a 130-item timed test of fluid intelligence, with separate verbal and nonverbal sections (21); the Watts-Vernon reading test, a test of reading comprehension requiring participants to select the appropriate words to complete 35 sentences (22); and a test of mathematical ability designed specifically for the study by the National Foundation for Educational Research in England and Wales. Scores on each of these tests were standardized and then summed to derive a total score of general cognitive ability standardized (to a mean of 0 and standard deviation of 1) in the analytical sample. Where missing at age of 15 years, values were imputed using standardized scores from similar cognitive tests undertaken at age of 11 years (*n* = 103) or 8 years (if missing at 15 and 11 (*n* = 48)) based on the observation that participants in the analytical sample with cognitive test scores at more than one age in childhood maintained a similar ranking over time (Pearson correlation coefficients between the cognitive score at age of 15 years and those at ages of 11 and 8 years were 0.87 and 0.72, respectively) (23).

### Covariates

Potential confounders from early life and factors in adulthood, which may mediate the main associations of interest, were selected a priori on the basis of previous findings in this study (4,6,7,14,24,25). The role of educational attainment and cognitive ability at age of 53 years, which may represent more proximal factors on a common pathway, were also investigated (11,12,26).

Birth weight, recorded to the nearest quarter pound, was extracted from birth records and converted into kilogram. Paternal occupation at age of 4 years (or at age of 11 or 15 years if missing at age of 4 years [*n* = 45]) was categorized using the Registrar General's Social Classification into the following three groups: high (I or II), middle (IIINM or IIIM), and low (IV or V). Maternal educational level reported during parental interviews in childhood was categorized into the following four groups: secondary and further or higher education; secondary only or primary and further or higher education; primary and further education (no qualifications attained); and primary education only. Own occupation at age of 53 years was categorized using the Registrar General's Social Classification into the same three groups as paternal occupation. Three behavioral risk factors (overweight or obesity, physical inactivity, and smoking) assessed at age of 53 years were combined in a cumulative behavioral risk factor score to create an ordinal scale ranging from 0 (most healthy) to 6 (least healthy). Information on the presence of four indicators of health status at age of 53 years (hand osteoarthritis, knee osteoarthritis, severe respiratory symptoms, and other disabling or life-threatening conditions) was combined to create a scale ranging from 0 (no reported health problems) to 4 (all 4 indicators reported). Derivation of these scores is described in detail elsewhere (14).

Educational level attained by age of 26 years was categorized into the following five groups: degree or higher; A levels, usually attained at age of 18 years, or their equivalents; O levels, usually attained at age of 16 years, or their equivalents; certificate of secondary education, clerical course or equivalent; and none. Two tests of cognitive ability at age of 53 years were included. Verbal memory was assessed using a 15-item word learning task with a total score representing the number of words correctly recalled over three trials (maximum: 45). Search speed was assessed using a timed letter search with the score representing the number of letters scanned in 1 minute (maximum score: 600).

### Statistical Analysis

Multinomial logistic regression models were used to test the associations of childhood cognitive ability with each of the different categories of change (versus the reference group) in grip strength and chair rise speed. Relative-risk ratios (RRRs) were estimated for a 1SD change in the childhood cognitive score with deviations from linearity formally tested to ensure assumptions of linearity were met. Initial models were adjusted for sex, after formally testing sex interactions. Subsequent models were adjusted in turn for potential confounders from early life (birth weight, paternal occupational class, and maternal educational level), potential adult mediators (own occupational class and cumulative scores of behavioral risk and health status), own educational level, and cognitive ability at age of 53 years (verbal memory and search speed) before all covariates were included in a fully adjusted model. Models were run on the sample with complete data on childhood cognitive ability and change (maximum number = 1954 [1811 for grip strength and 1875 for chair rise speed]). To maintain statistical power and minimize the level of bias introduced due to missing information, missing values of the covariates in this sample (birth weight [*n* = 5], paternal occupational class [n = 28], maternal educational level [n = 115], own educational level [n = 58], own occupational class [n = 4], behavioral risk score [n = 9], health indicator count [n = 25], verbal memory [n = 26], and search speed [n = 12]) were imputed using multiple imputation chained equations implemented in Stata Version 14. All analyses were run across 20 multiply imputed data sets and estimates were combined using Rubin's rules. Sensitivity analyses were performed to test the main associations in the maximum available samples with complete data (for comparison with those run on imputed datasets).

## RESULTS

One in five (20%) participants were classified as having experienced potentially meaningful decline in grip strength and in chair rise speed between ages of 53 and 60–64 years. An additional 5% were classified as stable high and a similar proportion as stable low (Table 1). Mean levels of performance at ages of 53 and 60–64 years in each of these categories are shown in Table S2, Supplemental Digital Content 1 (http://links.lww.com/PSYMED/A396). Distributions of each covariate are presented in Table 1.

**TABLE 1:**
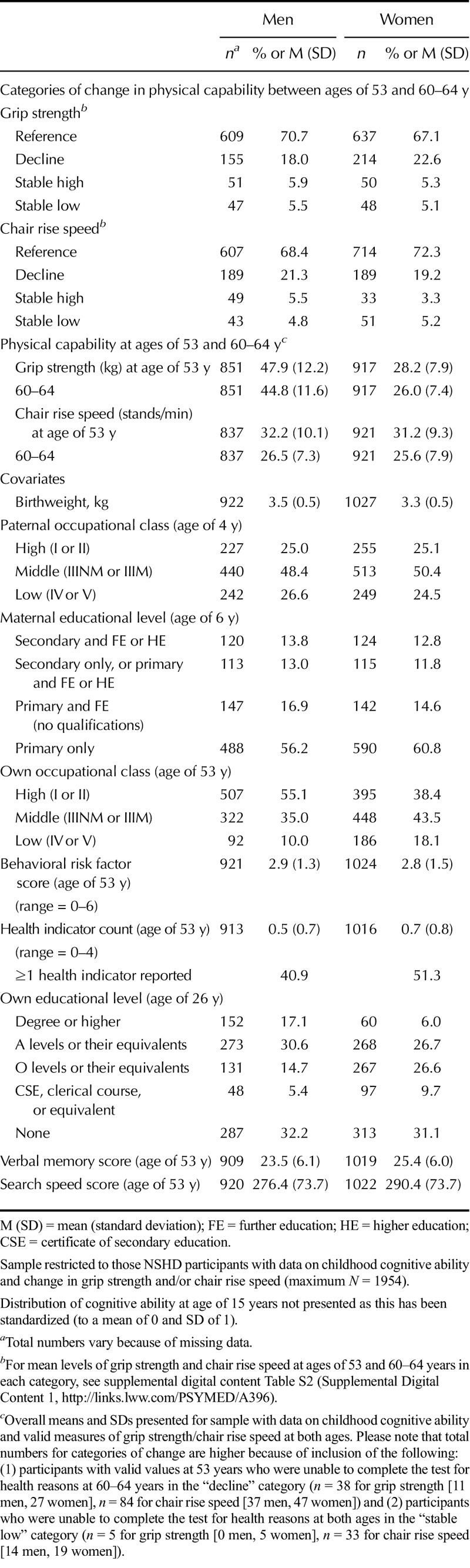
Characteristics of the Medical Research Council National Survey of Health and Development

### Changes in Grip Strength

In a sex-adjusted model, higher childhood cognitive scores were associated with lower risk of decline in grip strength (RRR of decline [versus reference] per 1SD increase in cognitive ability = 0.82, 95% confidence interval [CI] = 0.73–0.92) (Table 2). There was no evidence of sex interaction or deviation from linearity. Adjustment for each set of covariates, except educational level attained, had an impact on this association. Consistent with the finding that higher childhood socioeconomic position (indicated by paternal occupational class and maternal educational level) and higher verbal memory scores at age of 53 years were associated with lower risk of decline in grip strength (Table S3, Supplemental Digital Content 1, http://links.lww.com/PSYMED/A396), adjustment for these covariates had the greatest impact. In a fully adjusted model, the main association was attenuated (RRR of decline [versus reference] per 1SD increase in cognitive ability = 0.90, 95% CI = 0.75–1.07). There was no clear evidence of associations between childhood cognitive scores and likelihood of being in the stable high or stable low categories of grip strength (versus reference), although associations with stable low were in the expected direction.

**TABLE 2:**
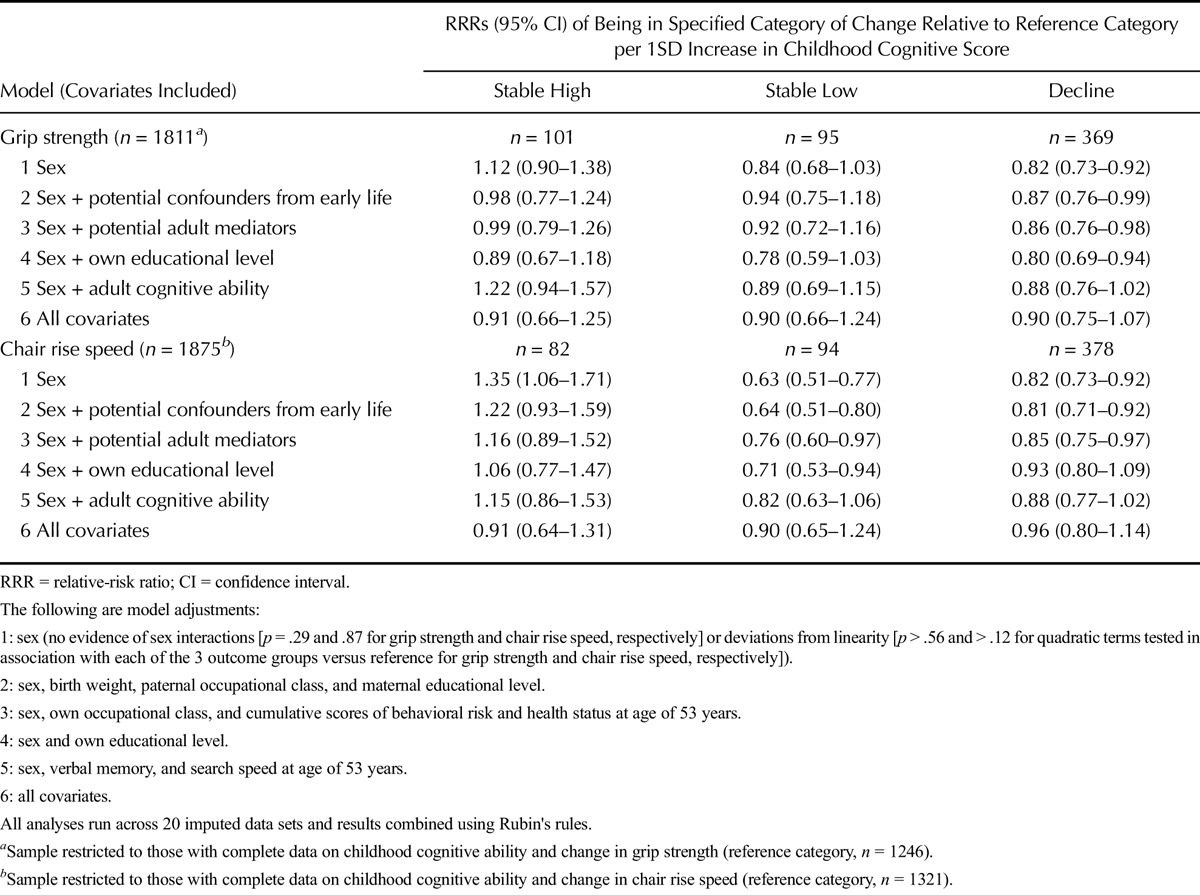
Associations of Childhood Cognitive Ability With Changes in Grip Strength and Chair Rise Speed Between Ages of 53 and 60–64 Years

### Changes in Chair Rise Speed

In a sex-adjusted model, higher childhood cognitive scores were associated with reduced risks of being in the decline and stable low categories of chair rise speed and with increased likelihood of being in the stable high category when compared with the reference category (Table 2). There was some evidence to suggest that associations with childhood cognitive scores were stronger for the stable low category (RRR = 0.63, 95% CI = 0.51–0.77) than the decline category (RRR = 0.82, 95% CI = 0.73–0.92). There was no evidence of sex interactions or deviations from linearity. Consistent with the finding that own educational level and verbal memory scores at age of 53 years were most strongly associated with changes in chair rise speed (Table S3, Supplemental Digital Content 1, http://links.lww.com/PSYMED/A396), adjustment for these covariates had the greatest impact on the associations between childhood cognitive scores and changes in chair rise speed.

There were no differences in findings when models were rerun on the maximum available samples with complete data.

## DISCUSSION

Higher childhood cognitive scores were associated with reduced risks of decline in grip strength and chair rise speed between ages of 53 and 60–64 years in a British birth cohort study. Participants with higher childhood cognitive scores were also less likely to be categorized as having stable low chair rise speed and more likely to be categorized as stable high.

The association between childhood cognitive ability and decline in grip strength was partially attenuated after adjustment for each set of covariates except educational level attained. Conversely, adjustment for educational level attained had a greater impact on the associations of childhood cognitive ability with change in chair rise speed than adjustments for other covariates. This suggests that different pathways (outlined hereinafter) may be implicated in explaining the observed associations of childhood cognitive ability with changes in grip strength and chair rise speed.

### Comparison With Other Studies

These new findings are consistent with those from previous studies showing links between higher cognitive ability in earlier life and better performance in different objective tests of physical capability assessed at a single time point (4,6–10). Our findings are also consistent with those from a recent study of Finnish males, which found that higher cognitive scores at a mean age of 20.3 years were associated with better self-reported physical functioning at a mean age of 71.4 years via an association with better self-reported physical functioning approximately a decade earlier (27). Our findings extend these previous analyses by demonstrating evidence of associations of childhood cognitive ability with changes in objective measures of physical capability in late midlife.

When lifetime cognitive performance was related to grip strength and chair rise speed at age of 53 years in the NSHD, no clear patterns of association were found with grip strength (4). As associations had been shown in older study populations, it was proposed that our null findings might be explained by an influence of aging processes not yet detectable at age of 53 years; our new findings support this. This also explains why associations were found with risk of decline but not with either of the other outcomes (i.e., stable low or high, associations that would be driven by an influence on baseline levels at age of 53 years). As associations with cognitive ability at age of 15 years were found with chair rise speed at age of 53 years (4), our finding of associations with all three categories of change is therefore consistent. At age of 53 years, associations of chair rise speed with markers of adult fluid ability (assessed by verbal memory and search speed tests) were stronger than those with markers of general cognitive ability (including the measure at age of 15 years), which is consistent with our finding of an attenuation of effect after adjustment for verbal memory.

### Explanation of Findings

The generation of maximal muscle force and chair rising performance both have a neurocognitive component; good performance on these tests, and the maintenance of this performance over time, depends to a certain extent on the healthy functioning of the central and peripheral nervous systems and the sensorimotor system (28–30). Measures that capture the functioning of particular aspects of these systems, such as cognitive tests, would therefore be expected to be associated with tests of physical capability, when measured contemporaneously. However, why these associations extend across life requires further explanation as follows.

A number of pathways linking childhood cognitive ability to later health outcomes have been proposed (13), some of which may contribute to explaining our observed associations with age-related declines in physical capability. For example, it has been suggested that childhood cognitive ability may predict health behaviors, health literacy, and entry into safe environments in adulthood. This is consistent with the partial attenuation of our observed associations after adjustment for behavioral risk factors and occupational class. That educational level attained attenuated associations between childhood cognitive ability and chair rise speed could be explained by education acting in concert with cognition on these extrinsic pathways. In previous analyses, these pathways have been shown to be more strongly related to chair rise speed than grip strength in midlife (31,32), which may explain the differences in the impact of these adjustments between outcomes.

It has also been suggested that childhood cognitive ability may be associated with later life outcomes because it acts as a marker of relevant underlying neurophysiological processes. Recent empirical evidence of associations between the development and subsequent degeneration of specific brain structures (33) and the finding that cross-sectional associations between cognitive ability and brain cortical thickness in old age were largely explained by childhood cognitive ability when this was taken into account (34) both lend support to the suggestion that these processes are lifelong. Related to which are systematic review findings confirming a link between whole brain volume and white matter volume and measures of physical capability (35).

Evidence suggests that higher childhood cognitive ability and greater educational attainment promote better midlife cognitive function (12). This may then confer benefits on physical capability as well as cognitive outcomes in later life. For example, in a French study of adults aged 65 to 85 years, associations between white matter lesions and slower walking speed were only found among participants with lower levels of education (36). The attenuation of our associations after adjustment for verbal memory at age of 53 years suggests that pathways linking childhood and adult cognition are likely to be important in explaining associations between childhood cognitive ability and age-related changes in physical capability, as found for mortality (26).

### Methodological Considerations

Key strengths of our analyses include availability of prospectively ascertained data on childhood cognitive ability, captured before premorbid decline, and on change in two objective measures of physical capability, which are components of common age-related disorders including frailty and sarcopenia and have been related to risk of premature mortality, mobility disability, and chronic disease (37–39). Analyses also benefited from the prospective ascertainment of a range of covariates. However, including factors such as education and adult cognitive ability could be viewed as an over adjustment.

Our method of analyzing change in physical capability was chosen with the aim of avoiding some of the limitations associated with modeling change continuously when data are only available at two time points, including regression to the mean, measurement error, and practice effects (19). We acknowledge that this chosen method also has important limitations and so our results need to be interpreted with caution; for example, there is heterogeneity between individuals within each of the four outcome groups for both measures (see Table S4, Supplemental Digital Content 1, http://links.lww.com/PSYMED/A396), especially the reference categories, which include relatively sizeable groups who seem to have improved over time (see Table S1, Supplemental Digital Content 1, http://links.lww.com/PSYMED/A396), and the “stable” groups where declines in means were observed (see supplemental digital content Tables S2 and S4, http://links.lww.com/PSYMED/A396). However, by choosing to analyze four outcome groups rather than using one of the established methods of creating binary categorizations of change scores (40), we hope to have taken greater account of the observed heterogeneity in intraindividual change (14). We have also identified different groups likely to be meaningful in the context of healthy aging similar to those identified in other recent studies using alternative approaches (41). Although the validity of our approach has not been formally assessed, there are clear differences in the levels of change in the mean grip strength and chair rise speed observed in these groups between ages of 53 and 60–64 years (see supplemental digital content Tables S2 and S4, http://links.lww.com/PSYMED/A396). Furthermore, additional analyses confirm that the four categories of change in grip strength and chair rise speed distinguish between groups of individuals with different health and disability prospects (see Table S5, Supplemental Digital Content 1, http://links.lww.com/PSYMED/A396).

The NSHD was selected at birth to be nationally representative and at ages of 53 and 60–64 years remained so in many respects (42,43). However, only those participants who were assessed at ages of 53 and 60–64 years could be included in our analyses and so losses to follow-up before age of 53 years are a potential source of bias in our analyses. Furthermore, bias may have been introduced by excluding participants assessed at age of 53 years who subsequently died; those participants in the NSHD who had weaker grip strength and slower chair rise speed at 53 had higher rates of all-cause mortality when followed up to age of 66 years (44). However, in a Finnish study that examined the impact of right censoring due to death, there was little evidence that estimates of annual change in grip strength before age of 65 years were altered (45) suggesting that any bias introduced by this in our analyses is likely to be minimal. Bias may also have been introduced by the exclusion of participants with missing data on childhood cognitive ability. However, there were no significant differences in the patterns of change when those participants with missing data on childhood cognitive ability were compared with those included in analyses. Implementing multiple imputation for missing data on covariates minimized another potential source of bias.

## CONCLUSIONS

Higher childhood cognitive ability was associated with reduced risks of decline in grip strength and chair rise speed during midlife. This suggests that additional insights may be provided by considering neurodevelopmental as well as neurodegenerative pathways when future research is undertaken to identify opportunities to prevent or minimize age-related declines in physical capability.

## Supplementary Material

SUPPLEMENTARY MATERIAL

## References

[1] Rosso AL Studenski SA Chen WG Aizenstein HJ Alexander NB Bennett DA Black SE Camicioli R Carlson MC Ferrucci L Guralnik JM Hausdorff JM Kaye J Launer LJ Aging, the central nervous system, and mobility. J Gerontol A Biol Sci Med Sci 2013;68:1379–86. 23843270 10.1093/gerona/glt089 PMC3805295

[2] Sorond FA Cruz-Almeida Y Clark DJ Viswanathan A Scherzer CR De Jager P Csiszar A Laurienti PJ Hausdorff JM Chen WG Ferrucci L Rosano C Studenski SA Black SE Lipsitz LA Aging, the central nervous system, and mobility in older adults: neural mechanisms of mobility impairment. J Gerontol A Biol Sci Med Sci 2015;70:1526–32. 26386013 10.1093/gerona/glv130 PMC4643615

[3] Anton SD Woods AJ Ashizawa T Barb D Buford TW Carter CS Clark DJ Cohen RA Corbett DB Cruz-Almeida Y Dotson V Ebner N Efron PA Fillingim RB Foster TC Gundermann DM Joseph AM Karabetian C Leeuwenburgh C Manini TM Marsiske M Mankowski RT Mutchie HL Perri MG Ranka S Rashidi P Sandesara B Scarpace PJ Sibille KT Solberg LM Someya S Uphold C Wohlgemuth S Wu SS Pahor M Successful aging: advancing the science of physical independence in older adults. Ageing Res Rev 2015;24:304–27. 26462882 10.1016/j.arr.2015.09.005 PMC4661112

[4] Kuh D Cooper R Hardy R Guralnik J Richards M, Musculoskeletal Study Team. Lifetime cognitive performance is associated with midlife physical performance in a prospective national birth cohort study. Psychosom Med 2009;71:38–48. 19124616 10.1097/PSY.0b013e31818a1620 PMC2890312

[5] Ferrucci L Cooper R Shardell M Simonsick EM Schrack JA Kuh D Age-related change in mobility: perspectives from life course epidemiology and geroscience. J Gerontol A Biol Sci Med Sci 2016;71:1184–94. 26975983 10.1093/gerona/glw043 PMC4978365

[6] Kuh D Hardy R Butterworth S Okell L Richards M Wadsworth M Cooper C Sayer AA Developmental origins of midlife physical performance: evidence from a British birth cohort. Am J Epidemiol 2006;164:110–21. 16757569 10.1093/aje/kwj193

[7] Kuh D Hardy R Butterworth S Okell L Wadsworth M Cooper C Aihie Sayer A Developmental origins of midlife grip strength: findings from a birth cohort study. J Gerontol A Biol Sci Med Sci 2006;61:702–6. 16870632 10.1093/gerona/61.7.702

[8] Deary IJ Whalley LJ Batty GD Starr JM Physical fitness and lifetime cognitive change. Neurology 2006;67:1195–200. 17030752 10.1212/01.wnl.0000238520.06958.6a

[9] Starr JM Deary IJ Lemmon H Whalley LJ Mental ability age 11 years and health status age 77 years. Age Ageing 2000;29:523–8. 11191245 10.1093/ageing/29.6.523

[10] Meincke RH Osler M Mortensen EL Hansen AM Is intelligence in early adulthood associated with midlife physical performance among Danish males? J Aging Health 2016;28:530–45. 26148944 10.1177/0898264315594139

[11] Richards M Shipley B Fuhrer R Wadsworth ME Cognitive ability in childhood and cognitive decline in mid-life: longitudinal birth cohort study. BMJ 2004;328:552. 14761906 10.1136/bmj.37972.513819.EE PMC381045

[12] Richards M Sacker A Lifetime antecedents of cognitive reserve. J Clin Exp Neuropsychol 2003;25:614–24. 12815499 10.1076/jcen.25.5.614.14581

[13] Deary IJ Whiteman MC Starr JM Whalley LJ Fox HC The impact of childhood intelligence on later life: following up the Scottish mental surveys of 1932 and 1947. J Pers Soc Psychol 2004;86:130–47. 14717632 10.1037/0022-3514.86.1.130

[14] Cooper R Muniz-Terrera G Kuh D Associations of behavioural risk factors and health status with changes in physical capability over 10 years of follow-up: the MRC National Survey of Health and Development. BMJ Open 2016;6:e009962. 10.1136/bmjopen-2015-009962 PMC4838696 27091818

[15] LaCroix AZ Guralnik JM Berkman LF Wallace RB Satterfield S Maintaining mobility in late life. II. Smoking, alcohol consumption, physical activity, and body mass index. Am J Epidemiol 1993;137:858–69. 8484377 10.1093/oxfordjournals.aje.a116747

[16] Stuck AE Walthert JM Nikolaus T Bula CJ Hohmann C Beck JC Risk factors for functional status decline in community-living elderly people: a systematic literature review. Soc Sci Med 1999;48:445–69. 10075171 10.1016/s0277-9536(98)00370-0

[17] Wadsworth M Kuh D Richards M Hardy R Cohort Profile: The 1946 National Birth Cohort (MRC National Survey of Health and Development). Int J Epidemiol 2006;35:49–54. 16204333 10.1093/ije/dyi201

[18] Kuh D Pierce M Adams J Deanfield J Ekelund U Friberg P Ghosh AK Harwood N Hughes A Macfarlane PW Mishra G Pellerin D Wong A Stephen AM Richards M Hardy R Cohort profile: updating the cohort profile for the MRC National Survey of Health and Development: a new clinic-based data collection for ageing research. Int J Epidemiol 2011;40:e1–9. 21345808 10.1093/ije/dyq231 PMC3043283

[19] Singer JD Willett JB Applied Longitudinal Data Analysis: Modeling Change and Event Occurrence, 1 ed Oxford: Oxford University Press; 2003.

[20] Fitzmaurice G A conundrum in the analysis of change. Nutrition 2001;17:360–1. 11369183 10.1016/s0899-9007(00)00593-1

[21] Heim AW The AH4 Group Test of Intelligence, NFER-Nelson: Windsor; 1970.

[22] Pigeon DA Details of the fifteen year tests. In: Douglas JWB Ross JM Simpson HR, editors. All Our future. London: Davies; 1968.

[23] Richards M Kuh D Hardy R Wadsworth M Lifetime cognitive function and timing of the natural menopause. Neurology 1999;53:308–14. 10430419 10.1212/wnl.53.2.308

[24] Richards M Hardy R Kuh D Wadsworth ME Birthweight, postnatal growth and cognitive function in a national UK birth cohort. Int J Epidemiol 2002;31:342–8. 11980795

[25] Richards M Hardy R Wadsworth ME Does active leisure protect cognition? Evidence from a national birth cohort. Soc Sci Med 2003;56:785–92. 12560011 10.1016/s0277-9536(02)00075-8

[26] Davis D Cooper R Terrera MG Hardy R Richards M Kuh D Verbal memory and search speed in early midlife are associated with mortality over 25 years of follow-up, independently of health status and early life factors. A British birth cohort study. Int J Epidemiol 2016;45:1216–25. 27498153 10.1093/ije/dyw100 PMC6639118

[27] Poranen-Clark T von Bonsdorff MB Tormakangas T Lahti J Wasenius N Raikkonen K Osmond C Salonen MK Rantanen T Kajantie E Eriksson JG Intellectual ability in young adulthood as an antecedent of physical functioning in older age. Age Ageing 2016;45:727–31. 27189726 10.1093/ageing/afw087 PMC5038877

[28] Clark BC Manini TM Special article “Green Banana”—Sarcopenia not equal dynapenia. J Gerontol A Biol Sci Med Sci 2008;63:829–34. 18772470 10.1093/gerona/63.8.829

[29] Manini TM Clark BC Dynapenia and aging: an update. J Gerontol A Biol Sci Med Sci 2012;67:28–40. 21444359 10.1093/gerona/glr010 PMC3260480

[30] Gonzalez-Freire M de Cabo R Studenski SA Ferrucci L The neuromuscular junction: aging at the crossroad between nerves and muscle. Front Aging Neurosci 2014;6:208. 25157231 10.3389/fnagi.2014.00208 PMC4127816

[31] Strand BH Cooper R Hardy R Kuh D Guralnik J Lifelong socioeconomic position and physical performance in midlife: results from the British 1946 birth cohort. Eur J Epidemiol 2011;26:475–83. 21416275 10.1007/s10654-011-9562-9 PMC3246593

[32] Cooper R Mishra GD Kuh D Physical activity across adulthood and physical performance in midlife: findings from a British Birth Cohort. Am J Prev Med 2011;41:376–84. 21961464 10.1016/j.amepre.2011.06.035 PMC3185208

[33] Douaud G Groves AR Tamnes CK Westlye LT Duff EP Engvig A Walhovd KB James A Gass A Monsch AU Matthews PM Fjell AM Smith SM Johansen-Berg H A common brain network links development, aging, and vulnerability to disease. Proc Natl Acad Sci U S A 2014;111:17648–53. 25422429 10.1073/pnas.1410378111 PMC4267352

[34] Karama S Bastin ME Murray C Royle NA Penke L Muñoz Maniega S Gow AJ Corley J Valdés Hernández MC Lewis JD Rousseau MÉ Lepage C Fonov V Collins DL Booth T Rioux P Sherif T Adalat R Starr JM Evans AC Wardlaw JM Deary IJ Childhood cognitive ability accounts for associations between cognitive ability and brain cortical thickness in old age. Mol Psychiatry 2014;19:555–9. 23732878 10.1038/mp.2013.64 PMC3998074

[35] Kilgour AH Todd OM Starr JM A systematic review of the evidence that brain structure is related to muscle structure and their relationship to brain and muscle function in humans over the lifecourse. BMC Geriatr 2014;14:85. 25011478 10.1186/1471-2318-14-85 PMC4105796

[36] Elbaz A Vicente-Vytopilova P Tavernier B Sabia S Dumurgier J Mazoyer B Singh-Manoux A Tzourio C Motor function in the elderly: evidence for the reserve hypothesis. Neurology 2013;81:417–26. 23803317 10.1212/WNL.0b013e31829d8761 PMC3776533

[37] Cooper R Kuh D Hardy R, Mortality Review Group, FALCon and HALCyon Study Teams. Objectively measured physical capability levels and mortality: systematic review and meta-analysis. BMJ 2010;341:c4467. 20829298 10.1136/bmj.c4467 PMC2938886

[38] Rantanen T Guralnik JM Foley D Masaki K Leveille S Curb JD White L Midlife hand grip strength as a predictor of old age disability. JAMA 1999;281:558–60. 10022113 10.1001/jama.281.6.558

[39] Cooper R Kuh D Cooper C Gale CR Lawlor DA Matthews F Hardy R Objective measures of physical capability and subsequent health: a systematic review. Age Ageing 2011;40:14–23. 20843964 10.1093/ageing/afq117 PMC3000177

[40] Frerichs RJ Tuokko HA A comparison of methods for measuring cognitive change in older adults. Arch Clin Neuropsychol 2005;20:321–33. 15797168 10.1016/j.acn.2004.08.002

[41] Ward RE Beauchamp MK Latham NK Leveille SG Percac-Lima S Kurlinski L Ni P Goldstein R Jette AM Bean JF A novel approach to identifying trajectories of mobility change in older adults. PLoS One 2016;11:e0169003. 28006024 10.1371/journal.pone.0169003 PMC5179086

[42] Wadsworth ME Butterworth SL Hardy RJ Kuh DJ Richards M Langenberg C Hilder WS Connor M The life course prospective design: an example of benefits and problems associated with study longevity. Soc Sci Med 2003;57:2193–205. 14512249 10.1016/s0277-9536(03)00083-2

[43] Stafford M Black S Shah I Hardy R Pierce M Richards M Wong A Kuh D Using a birth cohort to study ageing: representativeness and response rates in the National Survey of Health and Development. Eur J Ageing 2013;10:145–57. 23637643 10.1007/s10433-013-0258-8 PMC3637651

[44] Cooper R Strand BH Hardy R Patel KV Kuh D Physical capability in mid-life and survival over 13 years of follow-up: British birth cohort study. BMJ 2014;348:g2219. 24787359 10.1136/bmj.g2219 PMC4004787

[45] Stenholm S Harkanen T Sainio P Heliovaara M Koskinen S Long-term changes in handgrip strength in men and women—accounting the effect of right censoring due to death. J Gerontol A Biol Sci Med Sci 2012;67:1068–74. 22421705 10.1093/gerona/gls064

