# Supplementary material for: Childhood Cognitive Ability and Age-Related Changes in Physical Capability From Midlife: Findings From a British Birth Cohort Study
Source: Psychosom Med. 2017 Jun 9;79(7):785–91. doi: 10.1097/PSY.0000000000000482 (PMC5580377; doi:10.1097/PSY.0000000000000482)
Supplement: SUPPLEMENTARY MATERIAL [file psm-79-785-s001.docx]

**Supplemental digital content**

**Table S1:** Cross-tabulation of sex-specific standard deviation scores for grip strength and chair rise speed at ages 53 and 60-64 years used to identify four main outcome groups for each measure

| **Grip strength (G)/chair rise speed (C) SD score at age:** | **60-64 years** | | |
| --- | --- | --- | --- |
| **53 years** | < - 1SD | -1 SD to 1 SD | > 1SD |
| < - 1SD | **4.** Stable low  G:90; C:61 | **1.** Reference  G:156; C:147 | **1.** Reference  G:23; C:8 |
| -1 SD to 1 SD | **2.** Decline  G:180; C:146 | **1.** Reference  G:906; C:1039 | **1.** Reference  G:161; C:127 |
| > 1SD | **2.** Decline  G:14; C:5 | **2.** Decline  G:137; C:143 | **3.** Stable high  G:101; C:82 |

Note: G and C are the number of participants in each cell when the categorised sex-specific standard deviation scores for grip strength (G) and chair rise speed (C) at ages 53 and 60-64 years are cross-tabulated among the sample with data on childhood cognitive ability

Total Ns used in analyses are higher than those presented in table S1 due to additional inclusion of: (1) participants with valid values at 53 who were unable to complete the test for health reasons at 60-64 years in the ‘decline’ category (n=38 for grip strength, n=84 for chair rise speed) and; (2) participants who were unable to complete the test for health reasons at both ages in the ‘stable low’ category (n=5 for grip strength, n=33 for chair rise speed)

**Table S2:** Mean levels of grip strength and chair rise speed at ages 53 and 60-64 years in each category of change in the MRC National Survey of Health and Development (sample restricted to those with data on childhood cognitive ability)

|  | **Men** | | | **Women** | | |
| --- | --- | --- | --- | --- | --- | --- |
|  | **N^a^ (%)** | **Mean (SD)** | | **N^a^ (%)** | **Mean (SD)** | |
|  |  | **at 53y** | **at 60-64y** |  | **at 53y** | **at 60-64y** |
| **Grip strength (kg)**  Reference  Decline  Stable high  Stable low | 609 (70.7)  155 (18.0)  51 (5.9)  47 (5.5) | 46.1 (9.7)  53.7 (12.1)  68.2 (6.6)  28.7 (6.4) | 46.5 (8.4)  36.0 (11.6)  64.8 (7.3)  27.4 (4.3) | 637 (67.1)  214 (22.6)  50 (5.3)  48 (5.1) | 26.6 (5.9)  31.5 (8.4)  42.2 (8.7)  16.7 (2.5) | 27.3 (5.7)  20.3 (6.6)  39.1 (4.1)  15.6 (2.6) |
|  |  |  |  |  |  |  |
| **Chair rise speed (stands/min)**  Reference  Decline  Stable high  Stable low | 607 (68.4)  189 (21.3)  49 (5.5)  43 (4.8) | 29.8 (6.1)  36.5 (13.6)  51.0 (7.8)  18.8 (2.4) | 26.9 (5.4)  21.5 (5.8)  42.3 (6.4)  16.6 (2.0) | 714 (72.3)  189 (19.2)  33 (3.3)  51 (5.2) | 29.1 (6.0)  36.1 (12.9)  50.5 (7.9)  17.9 (2.9) | 26.1 (6.7)  21.5 (6.9)  42.5 (8.6)  15.2 (2.5) |

a. Total N in each category. Ns for presented means in some categories vary due to inclusion of: (1) participants with valid values at 53 who were unable to complete the test for health reasons at 60-64 years in the ‘decline’ category (n=38 for grip strength (11 men, 27 women), n=84 for chair rise speed (37 men, 47 women)) and; (2) participants who were unable to complete the test for health reasons at both ages in the ‘stable low’ category (n=5 for grip strength (0 men, 5 women), n=33 for chair rise speed (14 men, 19 women))

For mean values of grip strength and chair rise speed in each category estimated using the maximum available samples, please see Cooper et al 2016(1)

**Table S3:** Sex-adjusted associations* of each covariate with categories of change in grip strength and chair rise speed in the MRC National Survey of Health and Development (sample restricted to those with complete data on childhood cognitive ability and change (Max N=1811 for grip strength and 1875 for chair rise speed) though Ns vary due to missing data on covariates)

|  | **Relative-risk ratios (95% CI) of being in specified category of change relative to reference category** | | | | | | |
| --- | --- | --- | --- | --- | --- | --- | --- |
|  | **Grip strength** | | |  | **Chair rise speed** | | |
|  | **Stable high** | **Stable low** | **Decline** |  | **Stable high** | **Stable low** | **Decline** |
| Birth weight *per 1kg increase* | 1.75 (1.16, 2.64) | 0.50 (0.33, 0.76) | 1.03 (0.81, 1.30) |  | 0.98 (0.62, 1.55) | 1.12 (0.73, 1.72) | 1.03 (0.81, 1.30) |
|  |  |  |  |  |  |  |  |
| Paternal occupational class *I or II*  *III*  *IV or V* | 1.00  0.85 (0.53, 1.35)  0.60 (0.33, 1.10) | 1.00  1.37 (0.79, 2.37)  1.66 (0.91, 3.04) | 1.00  1.38 (1.01, 1.86)  1.70 (1.21, 2.38) |  | 1.00  0.42 (0.25, 0.70)  0.37 (0.20, 0.70) | 1.00  1.56 (0.89, 2.75)  1.43 (0.76, 2.70) | 1.00  1.20 (0.91, 1.60)  0.96 (0.68, 1.34) |
|  |  |  |  |  |  |  |  |
| *per category change* | 0.78 (0.58, 1.05) | 1.28 (0.95, 1.73) | 1.30 (1.10, 1.54) |  | 0.56 (0.40, 0.77) | 1.17 (0.87, 1.58) | 0.98 (0.83, 1.15) |
|  |  |  |  |  |  |  |  |
| Maternal educational level  *Secondary & FE or HE*  *Secondary only or, primary and FE or HE*  *Primary and FE (no qualifications)*  *Primary only* | 1.00  0.60 (0.29, 1.25)  0.83 (0.43, 1.58)  0.48 (0.28, 0.83) | 1.00  1.82 (0.65, 5.14)  2.23 (0.84, 5.93)  2.55 (1.08, 6.00) | 1.00  1.53 (0.92, 2.57)  1.75 (1.07, 2.87)  1.85 (1.22, 2.80) |  | 1.00  0.84 (0.36, 1.98)  1.22 (0.57, 2.58)  0.65 (0.34, 1.26) | 1.00  1.73 (0.65, 4.57)  1.34 (0.50, 3.60)  2.14 (0.96, 4.76) | 1.00  0.97 (0.60, 1.58)  1.17 (0.75, 1.83)  1.23 (0.85, 1.76) |
|  |  |  |  |  |  |  |  |
| *per category change* | 0.80 (0.68, 0.96) | 1.29 (1.03, 1.61) | 1.18 (1.05, 1.32) |  | 0.87 (0.71, 1.06) | 1.24 (1.00, 1.55) | 1.08 (0.97, 1.21) |
|  |  |  |  |  |  |  |  |
| Own occupational class *I or II*  *III*  *IV or V* | 1.00  0.55 (0.34, 0.88)  0.69 (0.36, 1.33) | 1.00  1.28 (0.80, 2.03)  1.50 (0.81, 2.77) | 1.00  1.40 (1.08, 1.81)  1.38 (0.97, 1.97) |  | 1.00  0.79 (0.49, 1.26)  0.33 (0.12, 0.93) | 1.00  1.46 (0.90, 2.37)  2.51 (1.41, 4.47) | 1.00  1.02 (0.80, 1.32)  1.32 (0.94, 1.86) |
|  |  |  |  |  |  |  |  |
| *per category change* | 0.73 (0.53, 1.00) | 1.24 (0.92, 1.66) | 1.22 (1.03, 1.44) |  | 0.68 (0.47, 0.97) | 1.57 (1.18, 2.09) | 1.12 (0.95, 1.32) |
|  |  |  |  |  |  |  |  |
| Own educational level *Degree or higher*  *A levels, or their equivalents*  *O levels, or their equivalents*  *CSE, clerical course or equivalent*  *None* | 1.00  1.10 (0.58, 2.08)  0.80 (0.39, 1.63)  0.34 (0.09, 1.22)  0.62 (0.31, 1.23) | 1.00  1.02 (0.47, 2.21)  1.42 (0.65, 3.13)  1.40 (0.53, 3.74)  1.08 (0.50, 2.30) | 1.00  1.42 (0.89, 2.27)  1.48 (0.91, 2.41)  1.94 (1.10, 3.43)  1.64 (1.04, 2.59) |  | 1.00  0.73 (0.39, 1.35)  0.52 (0.25, 1.07)  0.21 (0.05, 0.92)  0.37 (0.18, 0.76) | 1.00  0.99 (0.38, 2.61)  1.73 (0.67, 4.50)  3.58 (1.30, 9.87)  2.69 (1.11, 6.53) | 1.00  1.21 (0.77, 1.90)  1.19 (0.74, 1.92)  1.63 (0.92, 2.88)  1.93 (1.25, 2.99) |
|  |  |  |  |  |  |  |  |
| *per category change* | 0.84 (0.72, 0.98) | 1.02 (0.88, 1.19) | 1.09 (1.00, 1.19) |  | 0.77 (0.64, 0.91) | 1.34 (1.15, 1.57) | 1.18 (1.09, 1.28) |
|  |  |  |  |  |  |  |  |
| Verbal memory at age 53 *per 1SD increase* | 0.95 (0.78, 1.17) | 0.85 (0.69, 1.05) | 0.80 (0.71, 0.91) |  | 1.36 (1.07, 1.73) | 0.57 (0.46, 0.71) | 0.82 (0.73, 0.92) |
| Search speed at age 53 *per 1SD increase* | 1.08 (0.89, 1.32) | 1.03 (0.84, 1.27) | 1.01 (0.90, 1.13) |  | 1.34 (1.09, 1.64) | 0.72 (0.57, 0.91) | 0.96 (0.85, 1.08) |

* No evidence of interactions with sex when formally tested (Grip strength: birth weight p=0.90; father’s occupational class p=0.94; maternal educational level p=0.39; own educational level p=0.73; own occupational class p=0.91; verbal memory p=0.79; search speed p=0.41; Chair rise speed: birth weight p=0.26; father’s occupational class p=0.84; maternal educational level p=0.34; own educational level p=0.31; own occupational class p=0.60; verbal memory p=0.55; search speed p=0.09)

Deviations from linearity also formally tested but no evidence of this found.

Note: estimates for behavioural risk and health indicator count not shown as these have previously been reported in Cooper et al, 2016(1)

**Table S4:** Mean (SD) and inter-quartile range (IQR) of change scores in each outcome category of grip strength and chair rise speed

|  | **Men** |  |  | **Women** |  |
| --- | --- | --- | --- | --- | --- |
|  | **Mean (SD)** | **IQR** |  | **Mean (SD)** | **IQR** |
| **Grip strength (kg)**  Reference  Decline  Stable high  Stable low | 0.4 (11.1)  -18.7 (8.5)  -3.4 (8.6)  -1.2 (7.0) | -6.9 to 7.0  -22.8 to -13.1  -8.9 to -1.0  -4.8 to 2.7 |  | 0.7 (7.6)  -12.4 (5.8)  -3.1 (8.5)  -1.2 (3.8) | -4.5 to 4.8  -15.8 to -8.1  -4.6 to 1.3  -3.0 to 1.9 |
|  |  |  |  |  |  |
| **Chair rise speed (stands/min)**  Reference  Decline  Stable high  Stable low | -2.9 (7.1)  -16.6 (11.0)  -8.7 (10.9)  -2.2 (2.9) | -7.8 to 1.8  -21.1 to -9.6  -13.5 to -1.6  -4.0 to -0.1 |  | -3.0 (7.9)  -18.4 (8.4)  -8.0 (9.7)  -2.7 (3.2) | -7.8 to 1.1  -22.3 to -13.5  -11.8 to -3.6  -4.6 to -0.3 |

Note: Sample restricted to those with complete data on childhood cognitive ability and change. For Ns in each category please see table S2

Change scores calculated by subtracting value of grip strength (kg)/chair rise speed (stands/minute) achieved at 53 from value achieved at 60-64 whereby negative values reflect a decline with age.

**Table S5:** Proportion of study participants in each category of change in grip strength and chair rise speed who: 1) reported a long-term limiting illness, health problem or disability at age 60-64; 2) reported difficulty walking at age 60-64; 3) died between assessment at age 60-64 and age 70

|  | **% reporting long-term limiting illness, health problem or disability at age 60-64** | **% with self-reported difficulty walking at age 60-64** | **% who had died by age 70** |
| --- | --- | --- | --- |
| **Grip strength**  Reference  Decline  Stable high  Stable low  *p-value^a^* | 21  34  20  32  *<0.001* | 8  18  5  21  *<0.001* | 3  5  1  7  *0.004* |
|  |  |  |  |
| **Chair rise speed**  Reference  Decline  Stable high  Stable low  *p-value^a^* | 18  39  16  66  *<0.001* | 5  25  0  48  *<0.001* | 3  4  4  5  *0.57* |

^a^ p-values from chi-squared tests

Note: Sample restricted to those with complete data on childhood cognitive ability and change (N=1811 for grip strength and 1875 for chair rise speed). For Ns in each category please see table S2

**References**

(1) Cooper R, Muniz-Terrera G, Kuh D. Associations of behavioural risk factors and health status with changes in physical capability over 10 years of follow-up: the MRC National Survey of Health and Development. BMJ Open 2016;6:e009962.
